# ‘Under’ Surveillance: Impact of Race and Socioeconomic Status on Post-Treatment Breast Cancer Imaging

**DOI:** 10.1245/s10434-018-6418-5

**Published:** 2018-03-07

**Authors:** Lucy M. De La Cruz, Lawrence N. Shulman

**Affiliations:** 1 Department of Surgery, University of Pennsylvania, Philadelphia, PA USA; 2 Abramson Cancer Center, University of Pennsylvania, 3400 Civic Center Blvd, Philadelphia, PA 19104 USA

In patients with breast cancer, screening, diagnostics, treatment, and surveillance all contribute to their outcome. Disparities in cancer care and outcomes have been attributed to several patient-specific factors, such as age, ethnicity/race, and socioeconomic status, in a myriad of studies. Some have attributed this disparity to a lack of access to healthcare, delayed diagnosis, number of comorbidities, and worse tumor biology, all potentially leading to lower breast cancer survival. In particular, the transition between active treatment and post-treatment care has been identified as a place where patients can be vulnerable to failing to receive recommended care, not only relating to their cancer care but also in a patient’s general medical care such as diabetes monitoring, etc. This was highlighted in the Institute of Medicine’s 2005 report “*From Cancer Patient to Cancer Survivor: Lost in Transition*.”^1^ Few studies have looked at the impact of social and economic factors in the utilization of post-treatment imaging during the survivorship period. After breast cancer treatment, patients are at increased risk of local recurrence in the ipsilateral breast, and a twofold increased risk of a second breast cancer in either breast.^2^–^5^ Routine breast imaging has the goal of earlier detection of either of these events. Local recurrence is considered an independent predictor of survival, with increased relative risks of developing distant metastases or breast cancer-related deaths when compared with patients without a recurrence.^6^

In this issue, Adesoye and coauthors examine the patterns of utilization and factors associated with the use of screening mammography and magnetic resonance imaging (MRI) using the National Cancer Data Base (NCDB), as well as manual chart abstraction for a random sample of over 9000 patients who had undergone surgery for stages II and III breast cancer in 2006–2007.^7^ Patient charts were audited for the subsequent 4 years to determine the frequency of both mammography and breast MRI. By combining data from the NCDB, which contains patient demographics including age, socioeconomic status, race, ethnicity, comorbidities, stage and treatment, and chart abstraction, to most accurately determine imaging frequency, the authors have assembled one of the more robust databases to explore issues of post-treatment surveillance. They found that in year 1 after surgery, only 69.5% of patients underwent mammography, and by year 4 the rate had declined to 61%. Age (< 50 or > 80 years), race (Black), lack of insurance or government insurance, and low socioeconomic status were associated with underutilization of surveillance imaging. In addition, women with node-positive disease or larger tumors (> 5 cm) also had lower rates of post-treatment imaging. Some of these patient groups would be considered at higher risk for locoregional recurrences, and one might expect better post-treatment imaging surveillance rates. Of note, even in groups without these risk factors for underutilization, one-quarter of patients did not receive recommended surveillance imaging.

There was no difference in the use of imaging by hospital type, and academic teaching hospitals versus comprehensive community hospitals, versus community hospitals. A recent publication from the Commission on Cancer (CoC) and NCDB showed that risk-adjusted survival for stage III breast cancer varied by hospital type, with the best survivals at National Cancer Institute-designated comprehensive cancer centers and academic cancer centers, and poorer survival at community hospitals.^8^ Breast surveillance imaging does not appear to be a contributor to this disparity.

The indications for use of MRI for routine screening after breast cancer treatment remains indeterminate. The American Cancer Society guideline suggests that there is insufficient evidence to recommend or advise against annual MRI screenings for women with a personal history of breast cancer, while MRI screening is recommended as an adjunct to mammography for women with genetic mutations or women with more than a 20–25% lifetime risk of breast cancer.^9^ Most recommendations are based on consensus, not on randomized controlled trials, and interpretation of the breast MRI data from this study is uncertain. In this study, 12.5% of patients had MRIs performed in year 1, and 5.8% in year 4. The authors could not account for genetic or familial predisposition.

The fact that one-third of patients did not receive recommended post-treatment imaging is discouraging and worrisome. Although, as noted, women with certain demographics were more likely to not undergo recommended imaging, all groups had suboptimal rates of imaging. Schonberg et al. reported that 44% of patients treated for breast cancer experienced fear and anxiety about future mammograms, and whether this played into these data is unknown.^10^ Strategies and interventions aimed at eliminating barriers are needed and have significant public health implications in reducing healthcare disparities in breast cancer outcomes. The implementation of the Affordable Care Act (ACA) in 2010 was created, in part, to give cancer patients access to quality, affordable healthcare. All new health plans are required to cover preventive services, including mammography screening, at no cost to patients, and removed cost sharing for any preventive services covered by Medicare. Other programs such as the National Breast and Cervical Cancer Early Detection Program provide community-based breast screening to low-income, uninsured, and underinsured women; over 50% of their patients screened yearly are of racial/ethnic minority groups.

The creation of a nationwide standardized practice benchmark might increase utilization. This could be added to the survivorship care plan requirements for Commission on Cancer (CoC)-accredited cancer programs, with monitoring of compliance rates. For breast cancer patients, the survivorship visit should be used to establish the importance and schedule of post-treatment imaging, among other things. Institutions could use their electronic health records to create automated systems to identify patients who have not had post-treatment imaging at the recommended intervals.

Adesoye and colleagues have better defined a significant gap in post-treatment care for breast cancer patients, affecting all groups of patients but more pronounced in minorities and certain socioeconomic groups. It now rests on us as a profession to develop interventions to close this gap in care.
